# Amenability of an *Agrobacterium tumefaciens-*mediated shoot apical meristem-targeted *in planta* transformation strategy in Mango (*Mangifera indica* L.)

**DOI:** 10.1080/21645698.2022.2141014

**Published:** 2022-11-24

**Authors:** Kuldeep Pandey, Kesiraju Karthik, Sanjay Kumar Singh, Vinod, Rohini Sreevathsa, Manish Srivastav

**Affiliations:** a Division of Fruits and Horticultural Technology, ICAR-Indian Agricultural Research Institute, New Delhi, India; b ICAR-National Institute for Plant Biotechnology, Lal Bahadur Shastri Building, Pusa Campus, New Delhi, India; c Division of Genetics, ICAR-Indian Agricultural Research Institute, New Delhi, India

**Keywords:** *Agrobacterium tumefaciens*, Amrapali, apical meristem targeted *in planta* transformation, GFP, GUS, *Mangifera indica*, transgenics

## Abstract

Mango (*Mangifera indica* L.) is one of the most popular tropical fruits in the world owing to its rich taste, flavor, color, production volume and diverse end usage. Conventional mango breeding practices are unable to withstand the demand for improved varieties as it is time consuming and requires heavy investment. However, problems associated with traditional plant breeding can be curtailed through genetic transformation. Nevertheless, major limitation of transgenic development has been its recalcitrant nature toward tissue culture practices involving latent microbial infection, phenol exudation, etc. This opens wide scope for tissue culture-independent *in planta* transformation approaches These strategies have proved to be easy to execute and cost effective in producing large number of transformants. One such apical meristem targeted *in planta* approach was successfully exploited to demonstrate its utility in transforming a tree species. Mango variety Amrapali was transformed with two visual marker gene vectors *GFP::hptII* in pCAMBIA1302 and *GUS::nptII* in pCAMBIA2301 individually, to demonstrate its amenability. Preliminary confirmations identified 65.0% of *GFP* and 57.14% of *GUS* plants to be transformed. Further, molecular characterization of these primary transformants demonstrated transgene integration at genomic and transcript level in some of the plants. This established protocol holds good for functional gene validation and knock in/out studies and aid in mango improvement programs.

## 1. Introduction

Mango (*Mangifera indica* L.) belonging to family Anacardiaceae is an allopolyploid (2 n = 40) with medium genome size (~439 Mbp). It is the most widely grown fruit crop in India and acclaimed as “King of fruits.” India is one of the largest grower and exporter of mango, yielding foreign earnings of 39.6 million US dollars (https://www.statista.com/).^1^ In India, mango is being cultivated in an area of 2315 thousand hectares with annual production of 20899 thousand metric tonnes (https://nhb.gov.in/).^2^

Mango cultivation deals with biennial bearing habit, large tree size, susceptibility to major diseases (mango malformation, anthracnose, powdery mildew, bacterial black spot); pests (mango hopper, mealy bug, fruit fly, stone weevil); short-post-harvest life and physiological disorders (spongy tissue, jelly stone) being the major constraints.^3,4^ Conventional breeding of woody perennial fruit crops such as mango is difficult due to their long juvenile phase, existence of self-incompatibility, high degree of cross-pollination, low fruit set, high fruit drop, development of single seed per fruit, polyembryony, allopolyploid nature, highly heterozygous genetic background and lack of information about inheritance pattern of important quantitative traits.^4,5^ Moreover, improving popular mango cultivars by introducing genes from other wild species through interspecific hybridization has also been inadequate due to cross incompatibility barriers.^6^ Genetic transformation facilitates the introduction of a desired gene into the plant genome to overcome problems associated with traditional plant breeding.^7^

Mango micropropagation has not achieved much economic success than compared to other horticultural crops. This is due to several challenges that are associated with mango *in vitro* culture, including latent microbial infection, phenol exudation, culture medium discoloration, explant browning, *in vitro* recalcitrance of tissues either singly or in combination imperil the entire tissue culture attempts.^4^

Non-availability of *in vitro* regeneration protocols is mainly due to the basic barriers which involve excessive phenolic exudation post excision of explants (activation of oxidative enzyme system), explant browning (necrosis), culture media discolorations, deep-seated microbial contamination; slow and sporadic *in vitro* response of mango to tissue culture.^8^ Few studies have demonstrated genetic transformation of mango using *Agrobacterium tumefaciens*^6,9–11^ and gene gun^12^ with varying levels of success. Further, *in vitro* regeneration is genotype-dependent, time resilant and prone to somaclonal variations.^13,14^ Thus, to overcome the concerns associated with difficult-to-regenerate crops, the need of *in planta* approaches have begun to gain importance.^15,16^

Tissue culture-independent *in planta* transformation has been demonstrated in many crops such as *Brassica rapa*,^17^ *B. napus*,^18^ *B. campestris*,^19^ *Arabidopsis thaliana*,^20^ *Medicago truncatula*,^21^ *Raphanus sativus*,^22^ *Solanum lycopersicum*,^23^ *Glycine max*,^24^ *Melilotus alba*,^25^ *Zea mays*,^26^ *Oryza sativa*,^27^ *Citrus maxima*^28^ and *Passiflora edulis*.^29^ Several *in planta* transformation strategies have been developed using different tissues, *i.e*., seed, epicotyl, shoot apical meristem, flower, fruit etc.^30^

The advantages of *in planta* approaches are that they are cost effective, easy to execute and can produce a large number of transformants in a short period of time. Several reports have confirmed high transformation efficiencies in different crops.^31^ Among several *in planta* transformation techniques, apical meristem mediated transformation targets T-DNA to the growing shoot apical meristematic regions *in vitro* and allows the development of plants *ex vitro*. The methodology has been unequivocally proved in different crops like field bean,^32^ groundnut,^33^ capsicum,^34^ chili,^35^ pigeon pea,^36,37^ flax^38^ and cotton.^39,40^

Genetic transformation of mango holds significant potential, which can give leads in solving the problem of flowering, alternate bearing habit, development of parthenocarpy varieties and tolerance to different biotic and abiotic stresses. Furthermore, there is no information available on *in planta* transformation of mango. In the present study, our team has developed a successful strategy for transforming mango with the apical meristem-targeted *in planta* genetic transformation protocol. This strategy is expected to provide an alternate approach over tissue culture mediated transformation to develop genetically modified mango genotypes, which can hasten and shorten the varietal improvement programs.

## 2. Materials and Methods

### 2.1. Plant Material and Binary Vectors Used for Transformation

In the present study, mango variety Amrapali, was used for the development of transformants. Seeds were surface sterilized with Ridomil Gold® (Syngenta Basal, Switzerland), seed coat was removed to facilitate germination and sown in plastic bags containing sterile potting media (cocopeat, vermiculite and perlite, 3:1:1). These bags were maintained under controlled conditions (26 ± 1°C; RH 65–75%; 16/8 h photoperiod of 57 µmole m^−2^ s^−1^) till transformation. Two-week-old seedlings were used as explants for *Agrobacterium* infection.

**Figure 1.**
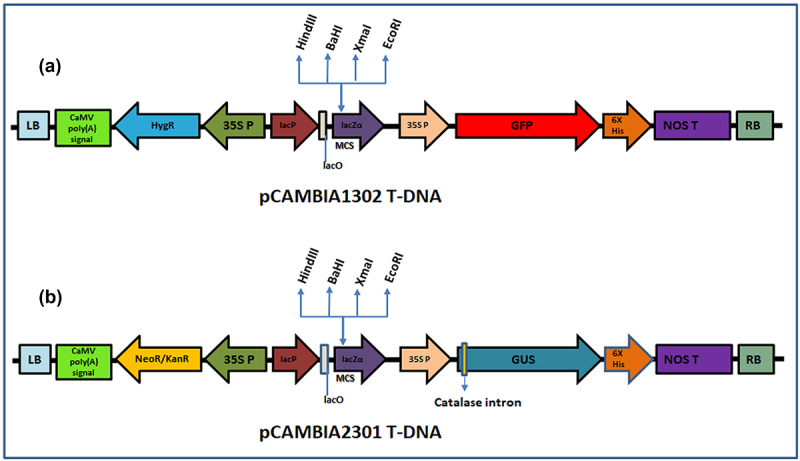
T-DNA of pCAMBIA1302 and pCAMBIA2301 vectors used for transformation. (a) T-DNA region of pCAMBIA1302 harboring *GFP* reporter and hygromycin resistance genes (b) T-DNA region of pCAMBIA2301 harboring *GUS* reporter and kanamycin resistance genes. LB; left border, CaMV poly (A) signal; cauliflower mosaic virus polyadenylation signal, HygR; Resistance to hygromycin; NeoR/KanR; Resistance to Kanamycin, 35SP; 35S promoter, lacP; lac promoter, lacO; lac operon, MCS; multiple cloning sites, NOS T; Nopaline synthase terminator, RB; right border.

*Agrobacterium tumefaciens* strain EHA105 harboring binary vectors pCAMBIA1302 carrying *GFP* (Green Fluorescent Protein) gene and *hptII* as antibiotic gene; pCAMBIA2301 carrying *GUS* (β-glucuronidase) marker gene which contains a 5’ extension of modified castor bean catalase intron (190 bp) to facilitate expression in plants but not in bacteria and *nptII* selectable marker gene were used for transformation of mango seedlings individually (Fig. 1a, b).

### 2.2. Development of Transgenics through an Apical meristem-targeted *in planta* Transformation Strategy

**Figure 2.**
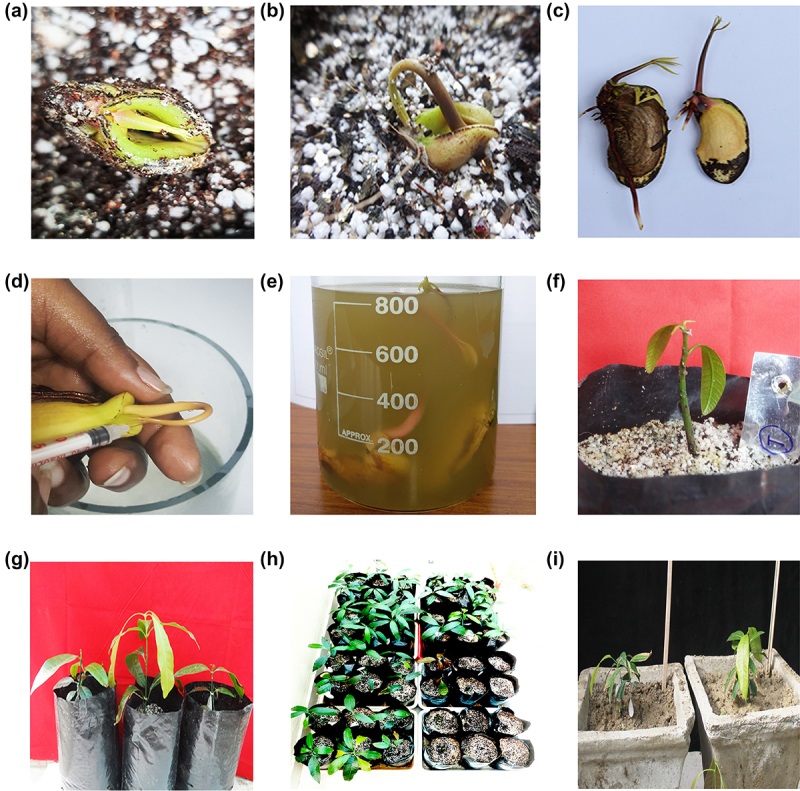
Strategy of tissue culture-independent *in planta* transformation in Mango (*Mangifera indica* L.). (a) Seeds without seed coat sown in potting mixture allowed to germinate; (b) Hook shaped plumule emerging out of seed indicating its growth; (c) Stage of seedling suitable for pricking; (d) Pricking of the emerging plumule with an insulin needle; (e) *Agrobacterium-*mediated transformation of seedlings; (f) Planting of transformed seedlings onto polybag and recovery of primary transformants; (g) Recovered plants in growth chamber (h) Plants transferred to glasshouse; and (i) established plants.

**Figure 3.**
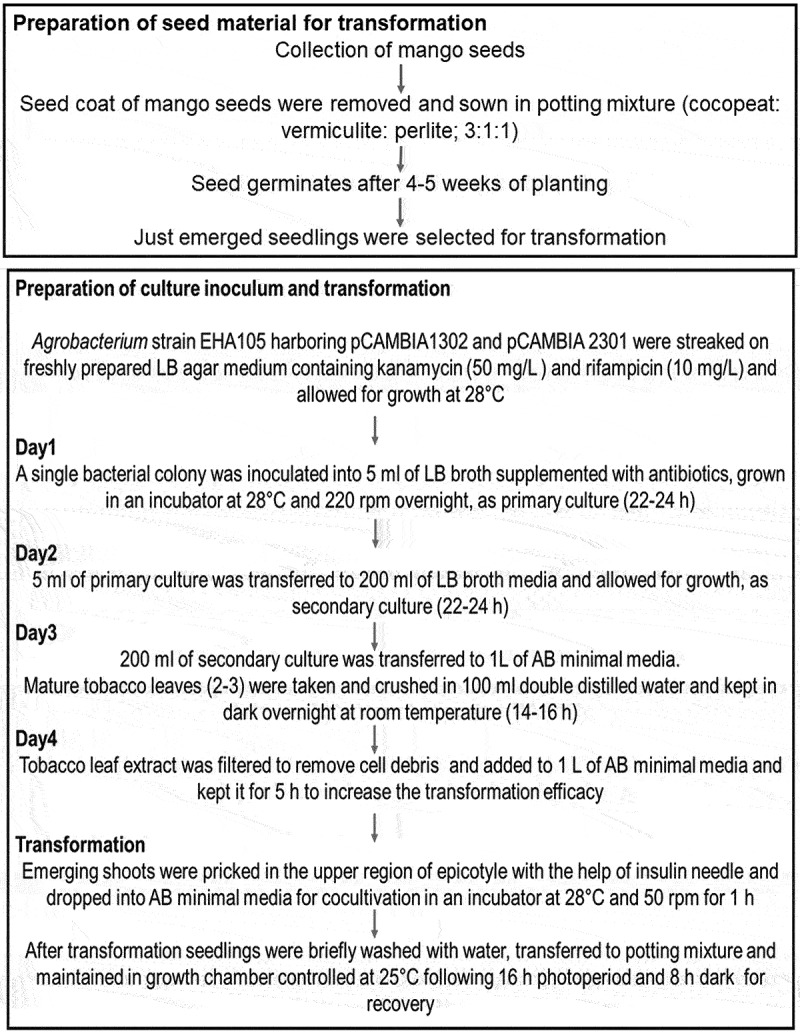
Schematic workflow of different steps involving *in planta* transformation in Mango.

Axenic culture of *Agrobacterium* harboring 35S::*GFP* and 35S::*hptII* in pCAMBIA1302 and 35S:: *GUS* and 35S:: *nptII* in pCAMBIA2301 from freshly streaked culture plate was inoculated into 5 ml LB medium (pH 7.0) containing 50 mg/L kanamycin, 10 mg/L rifampicin and incubated overnight at 28°C. The 5 ml starter culture on the next day was transferred to 200 ml of LB broth suplemeneted with antibiotics, which was later inoculated into 1 L of Winans’ AB minimal medium (pH 5.2)^41^ and incubated for 18 h at 28°C; 220 rpm. Two-week-old mango seedlings with emerging plumules were punctured 15–20 times with an insulin syringe at the apical meristem and incubated in AB minimal medium previously supplemented with crushed mature tobacco leaf extract^42^ maintained at 28°C; 50 rpm for 5 h. The plants were later allowed to grow under controlled conditions until they recovered from injury and resumed their growth (Figs. 2 and 3).

### 2.3. Identification of Putative Transformants

#### 2.3.1. GFP Expression

Seedlings transformed with pCAMBIA1302 were preliminarily confirmed using a fluorescence microscope with a 488 nm excitation wavelength. GFP expression in primary transformants, 36 h post infection was observed under a fluorescence microscope (ZEISS SteREO Discovery V20 microscope, Oberkochen, Germany). An excitation wavelength of 488 nm and 505–530 band-path filter (which permits visualization of GFP by blue light) to separate GFP and a 560 long-pass filter to determine chlorophyll fluorescence were used.^16^

#### 23.2 *GUS* Histochemical Analysis

Seedlings transformed with pCAMBIA2301 were initially confirmed by *GUS* histochemical analyses. Excised tissues (leaf and stem) after 76 h of *Agrobacterium* infection were incubated overnight in *GUS* assay buffer (0.1 M phosphate buffer, pH 7.0, 2 mM X-Gluc, 5 mM each of potassium ferricyanide and potassium ferrocyanide and 0.1% Triton X100) at 37°C in water bath. Chlorophyll present in the tissues were later destained using 75% ethanol (v/v).^43^ GUS expression at cellular level was observed using binocular microscope (Olympus, CX33, Shinjuku, Tokyo, Japan).^44^

### 2.4. Molecular Analyses of Transgenic Plants

#### 2.4.1. DNA Isolation

The leaves of transgenic and wild-type mango plants were crushed in liquid nitrogen to isolate genomic DNA using a CTAB (Cetyl Trimethyl Ammonium Bromide) method^44^ with minor modifications (added 1% PVP w/v for removal of phenols). For purification of DNA, 2 µl RNase A (10 mg/ml) was added per 200 µl of crude DNA solution and incubated for 1 h at 37°C, then treated with an equal volume of phenol: chloroform: isoamyl alcohol (25:24:1) and then precipitated with ethanol. The concentration and quality of DNA was estimated using NanoDrop™ (Thermo Scientific, Waltham, Massachusetts, USA) at 260 nm and by electrophoresis on 0.8% agarose gel.

#### 2.4.2. Polymerase Chain Reaction (PCR) Analyses

The presence of transgenes and *Agrobacterium* specific *VirD1* gene in the genome of putative mango transformants was assessed through PCR. The PCR reaction mixture (25 µl) containing 1 U *Taq* DNA polymerase (GeNie, Bengaluru, Karnataka, India), 1X assay buffer (10 mM pH 9.0 Tris–HCl, 50 mM KCl, 1.5 mM MgCl_2_, 0.01% gelatin), 2.5 µM of each dNTP, 0.5 µl of each forward and reverse primer (Table 1) at a final concentration of 10 pM and 100 ng of template DNA was used to amplify the transgenes. PCR amplification was carried out in a thermal cycler (Applied Biosystems® Veriti® 96-Well Fast Thermal Cycler, Waltham, Massachusetts, USA) programmed with a hot start of initial denaturation at 95°C for 5 min followed by 35 cycles of denaturation at 95°C for 1 min, annealing at 65°C for *GFP*, 55°C for *hptII* & *GUS*, 61°C for *VirD1* and 58°C for *nptII* for 1 min, extension at 72°C for 1 min, final extension at 72°C for 10 min. Amplified gene products of size 571 bp (*GFP*), 700 bp (*hptII*), 438 bp (*VirD1*) 750 bp (*nptII*) and 1 kb (*GUS*) were visualized by gel electrophoresis.

**Table 1.** List of primers used in the study.

Primer ID	Primer sequence (5′-3′)	Amplicon Size (bp)
**Primers used for PCR amplification**		
*GFP* FP	TGGGCACAAATTTTCTGTCAGTGGA	571 bp
*GFP* RP	ATGCCATGTGTAATCCCAGCAGCT	
*hptII* FP	GCTCGATACAAGCCAACCAC	700 bp
*hptII* RP	CGAAAAGTTCGACAGCGTCTC	
*GUS* FP	TTA TGC GGG CAA CGT CTG GTAT	1 kb
*GUS* RP	TGA CAA AAA CCA CCC AAG CGT	
*NptII* FP	CCGGAATTCATGATTGAACAA	750 bp
*NptII* RP	CCCAAGCTTCAGAAGAACTC	
*VirD1*FP	ATGTCGCAAGGCAGTAAGCCA	438 bp
*VirD1* RP	GGAGTCTTTCAGCATGGAGCAA	
**Primers used for sqRT-PCR**		
sqRT-PCR *GFP* FP	TCCACACAATCTGCCCTTTC	124 bp
sqRT-PCR *GFP* RP	CTATACAAAGCTAGCCACCACC	
sqRT-PCR *hptII* FP	GTCAGGCTCTCGCTAAACTC	130 bp
sqRT-PCR *hptII* RP	ATGTCCTGCGGGTAAATAGC	
sqRT-PCR *GUS* FP	ACCTCGCATTACCCTTACGCTG	122 bp
sqRT-PCR *GUS* RP	CCCGCTTCGAAACCAATG	
sqRT-PCR *NptII* FP	ATTGCACGCAGGTTCTCC	67 bp
sqRT-PCR *NptII* RP	TGTCTGTTGTGCCCAGTCA	
sqRT-PCR *MiACT1* FP	GTTTCCCAGTATTGTGGGTAGG	134 bp
sqRT-PCR *MiACT1* RP	AGATCTTTTCCATATCATCCCAGTT	

#### 2.4.3. Genomic Southern Analysis

For identification of T-DNA copy number in transgenic plants developed using both the binary vectors, 10 µg of genomic DNA from transgenic and wild-type plants was digested with *Hind*III (NEB high fidelity, New England Biolabs, Ipswich, Massachusetts, USA) overnight and separated on 0.8% agarose gel in 1X TAE buffer at constant voltage of 40 V. Restricted fragments were transferred onto a positively charged nylon membrane (Amersham™ Hybond™-N^+^) by capillary movement using 20× SSC and the membrane was later UV cross-linked. The membrane was hybridized with a DIG labeled 571 bp *GFP* and 750 bp *nptII* gene fragment for their corresponding transgenic plants. The blot was further processed with washing, blocking, and development as per manufacturer’s instructions (Roche Holding AG, Basel, Switzerland). The membranes were exposed to X-ray film for 1 h in dark and later observed for hybridization signal.

### 2.5. Analysis of Transgenic Plants for Transcript Accumulation by sqRT-PCR

Total RNA was isolated from transgenic and wild-type mango plants using a total RNA isolation kit (Spectrum^TM^, Sigma Aldrich, St. Louis, MO, United States). Further, the isolated RNA was quantified using Nanodrop™ 3300 (ThermoFisher Scientifc, Carlsbad Waltham, Massachusetts, USA) and transcribed to cDNA using SuperScript® (VILO^TM^, Invitrogen, Carlsbad, CA, USA). sqRT-PCR was performed with 100 ng of diluted cDNA as a template.

*M. indica* actin 1 (*MiACT1*) was used as an internal control gene.^45^ PCR reaction mixture of 25 µL consisting of 2.5 µL 10× *Taq* buffer, 10 pM each of forward and reverse primer, 2.5 µM dNTPs, and 1 U of Taq DNA polymerase (Bangalore Genei, Bengaluru, India); 1 µL of diluted cDNA was made up to a final volume of 25 µL with nuclease-free water (Invitrogen, Waltham, Massachusetts, USA) in a thermocycler (Applied Biosystems® Veriti® 96-Well Fast Thermal Cycler, Waltham, Massachusetts, USA). PCR program consisted of an initial denaturation step at 95°C for 4 min followed by 30 cycles of denaturation at 95°C for 30s, annealing at 58°C for 30s for *MiACT1* (134 bp), *nptII* (67 bp), *GUS* (111 bp), *GFP* (124 bp) and *hptII* (130 bp) gene RT-primers (Table 1) and extension at 72°C for 30s. The final extension was carried out at 72°C for 7 min to amplify specific gene products. “Blank” was devoid of cDNA, wild type contained 1 µL of cDNA of wild type, and positive control contained 25 ng of pCAMBIA2301/ pCAMBIA1302. The amplified gene products were analyzed on a 2.0% agarose gel.

## 3. Results and Discussion

Genetic transformation involves introduction of foreign genes to modify horticultural traits in perennial plants without changing their phenotype. Though mango genetic transformation has great potential,^46,47,48,11^ it is negatively affected by the non-availability of regeneration protocols and recalcitrance to tissue culture. Despite several attempts made by researchers to regenerate mango using leaf^49,50^ and shoots explants,^51,52^ it was proved inefficient and established mango in the category of hard to deal with tissue culture-based approaches. Unavailability of an efficient regeneration protocol makes transformation more difficult in mango. However, *in planta* transformation strategies provide an alternative to evade all the steps involved in tissue culture.^15,16^ The current study provides evidences for the development of transgenics in mango by an apical meristem targeted *in planta* transformation protocol.

### 3.1. Apical meristem-targeted in Planta Transformation

Two-week-old mango seedlings were subjected to transformation with pCAMBIA1302 and pCAMBIA2301 (Fig. 1a-b). The emerging shoots with brown-green coleoptiles were pricked in the upper region of the epicotyl, near the apical meristem with an insulin syringe (Fig. 2a-d). The punctured seedlings were co-cultivated with *Agrobacterium* strains (pCAMBIA1302/ pCAMBIA2301) in AB minimal medium containing wounded tobacco leaf (mature, yellowish-green) extract to increase the virulence of *Agrobacterium* (Fig. 2e). Pricked seeds were incubated on a rotatory shaker at 28^ο^C with 50 rpm for 2 h. After infection, seeds were thoroughly washed with autoclaved double distilled water and transferred to polybags containing sterilized growing media (cocopeat, vermiculite and perlite, 3:1:1 ratio) and maintained under diffused light initially and later transferred to direct light in growth chambers maintained at 25°C with 16 h light and 8 h dark photoperiod (fig. 2f). Diffused light has positive effect on the shoot regeneration and dark incubation was always found useful in regeneration and transformation experiments.^28,29,53,54^ Seedlings took 4–5 weeks for recovery during which, they were regularly irrigated with sterile-double distilled water and Hoagland solution (Fig. 2g). Recovered plants were later transferred to transgenic glasshouse as per regulatory guidelines (Fig. 2h, i). The overview of apical meristem targeted *in planta* transformation has been provided in a flow chart (Fig. 3).

Tissue culture mediated regeneration and transformation have been proven to be disadvantageous in several plant species due to low transformation efficiencies, genotype dependence and are time consuming.^55–57^ However, the *in planta* transformation approach does not demand any sterile growth conditions, phyto-hormones, is less time-consuming, needs low-cost inputs and also genotype independent.

### 3.2. Preliminary Screening of Transformants

Detection of visible scorable markers like *GUS* and *GFP* provide an early signal of transformability and/ or successful transformation of infected tissues.^38^ Therefore, in this study, GFP expression and GUS histochemical analysis were performed in their specific transformants to identify primary transformants.

**Figure 4.**
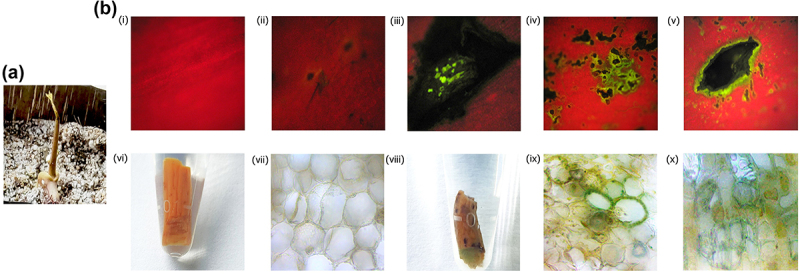
GFP expression and GUS histochemical analysis of primary transformants in (a) Recovered primary transformant exhibiting pricked sites (b) (i) Absence of GFP expression in wild-type seedlings, (ii) Pricked regions not exhibiting GFP fluorescence upon infection (iii–v) GFP expression identified in the pricked region in primary transformants of Mango. Absence of GUS expression in wild type (vi) shoot region (vii) cellular level. GUS expression (viii) in the shoot region (ix–x) at cellular level of primary transformants.

As *Agrobacterium* infection is a random event, GFP expression analysis in the pricked apical meristematic regions (Fig. 4a) identified GFP expression in some transformed seedlings (Fig. 4b iii–v) indicating that few of the infected seedlings were found to be positive toward the transformation strategy. Some transgenic plants lacked expression in the pricked regions indicating the chances of transformation as random (Fig. 4b ii). Further, *GFP* expression was absent in wild type seedlings (Fig. 4b i). Microscopic observation under UV illumination revealed that out of 40 seedlings taken for visualization, 26 seedlings displayed the presence of green fluorescent protein (GFP) and were selected as primary transformants (T0 plants) of which, 24 plants were transferred in transgenic glasshouse. This precisely demonstrates a preliminary confirmation of 65.0% of the seedlings having *GFP* expression and putatively transgenic (Table 2).

**Table 2.** Preliminary confirmation and percentage transformability of Mango using *in planta* transformation strategy.

Recovery			Preliminary confirmation			Molecular characterization			
No of seeds taken for transformation	No of plants Recovered	Percentage	No of plants	No of positive plants	Percentage	PCR			
						No of plants	No of positive plants	Percentage	Percentage chimeric plants produced
GFP- 70	55	78.57	40	26	65.0	24	13	54.16	18.57
GUS- 40	31	77.50	7	4	57.14	12	08	66.67	20.0

Another batch of plants where *GUS* gene was used as scorable marker identified primary transformants which demonstrated the presence of GUS expression in the pricked regions. Out of 30 primary transformants, GUS histochemical assay was performed using 7 plants and remaining plants were allowed to grow normally. It was found that out of 7 plants, 4 showed GUS expression at the pricked regions (Fig. 4b, viii). However, wild type plants did not show any color in stem and its dissections as they lacked the *GUS* transgene (Fig. 4b vi, vii). Presence of GUS expression at cellular level was further confirmed by observing the GUS-stained tissue sections under microscope (Fig. 4b ix, x). Since the binary vector used for transformation had a GUS gene with a catalase intron, the histochemical analysis provided evidences for the integration of the T-DNA into the genome of mango plants. Nearly 57% of the plants showed GUS expression. Based on post pricking recovery and initial evidences of transformability in mango, the remaining 15 seedlings were transferred to the transgenic glasshouse and were allowed for growth.

A successful genetic transformation system seeks an appropriate visualization marker gene for the identification of transgenic plants.^58^ In our experiment, *GFP* and *GUS* were used as screenable marker genes and similar studies have been reported in mango,^6^ pummelo,^28^ and passion fruit.^29^

### 3.3. Recovery of Primary Transformants

Recovery of plants after genetic transformation and their establishment is essential for the success of the protocol. After transferring plants to glasshouse, 24 of 26 *GFP* and 12 of 15 *GUS* plants could survive. These plants were transferred to pots filled with soil, sand and farm yard manure 2:1:1 (FYM) and allowed to grow in glasshouse. Plants were continuously irrigated and supplemented with Hoagland’s solution at regular intervals

### 3.4. Molecular Analyses for Transgene Integration in Mango

The purview of the study considering the perennial nature of mango (long juvenile phase of 6–8 years), deals with the demonstration of T-DNA integration in the T0 generation, despite the fact that the primary transformants produced through *in planta* transformation are chimeric. Similar kind of molecular characterization in the chimeric plants of other perennial tree species have been reported in pummelo,^28^ and passion fruit.^29^

**Figure 5.**
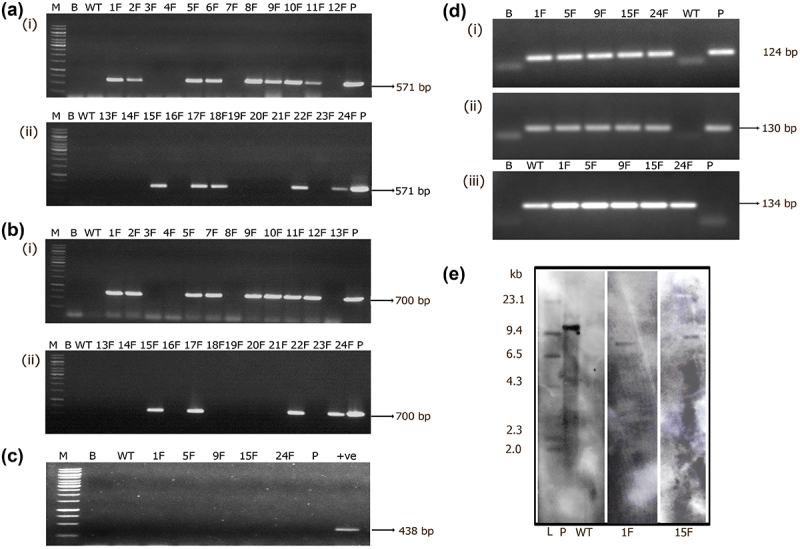
Molecular analysis of primary transformants harboring pCAMBIA1302 *GFP::hptII*. (a. i–ii; b. i–ii) PCR analysis for the amplification of *GFP* gene (571 bp) and *hptII* (700 bp) gene fragments. Lane M- 1Kb marker, Lane B- water blank, Lane WT- wild-type DNA, Lane P- plasmid (25 ng). Lanes 1 F-12 F and 13 F-24 F primary transformants. (c.) PCR analysis for the amplification of 438 bp *Agrobactrium-*specific *VirD1* gene in transgenic Mango plants. Lane M- 1 Kb marker (Thermo scientific), Lane B- water blank, Lane WT- wild type, Lanes 1 F, 5 F, 9 F, 15 F, 24 F- transgenic plants, Lane P- binary vector, +ve is DNA from *Agrobacterium* strain EHA 105 (d) sqRT-PCR analyses for the assessment of transgene transcripts. (i) 124 bp *GFP*, (ii) 130 bp *hptII* and (iii) 134 bp *MiACT1*. Lane B- water blank, Lane WT- wild type, Lanes 1 F, 5 F, 9 F, 15 F, 24 F- transgenic plants, Lane P- binary vector. (e) Genomic Southern analysis of transgenic plants probed with DIG-labeled 571 bp *GFP* gene fragment, Lane L- Lambda *Hind*III DNA digest, Lane WT- untransformed wild type, Lanes 1 F, 15 F- transgenic Mango, P- linearized plasmid of pCAMBIA1302 10 pg.

In order to confirm the presence of transgenes in the transgenic mango plants, genomic DNA was isolated from primary transformants and wild-type mango plants. PCR analysis was carried out using *GFP* and *hptII; GUS* and *nptII* gene-specific primers in their respective transgenic plants. Thirteen out of 24 plants showed the presence of 700 bp *hptII* and 570 bp *GFP* gene fragments (Fig. 5 Ai–ii, Bi–ii). Further, no amplification was found in wild type (WT) plants. The absence of *Agrobacterium* contamination in *GFP* transgenic plants were confirmed using PCR analysis of *VirD1* gene (Fig. 5c). This unequivocally demonstrated that the GFP expression in the transgenic plants was due to the integration of the transgene and not due to the persisting bacteria.

Transgenic plants that tested positive by PCR were further assessed at transcription level and expression of transgene was confirmed by semi-quantitative RT-PCR analysis. Five transgenic plants and positive control showed the amplification of 124 and 130 bp *GFP* and *hptII* gene transcript fragments in the respective transgenic plants. However, amplification was absent in wild type (WT) plants (Fig. 5d i–ii). An internal control (*MiACT1*) authenticated amplification of 134 bp fragment in both transgenic and wild-type plants (Fig. 5d, iii). Finally, transgenic mango plants were assessed to determine the copy number of the transgene. Two transgenic mango plants (1 F and 15 F) were found to have a single copy of the transgene integrated in the genome and the absence of hybridization signal in the wild type providing proof for transgene integration (Fig. 5e).

**Figure 6.**
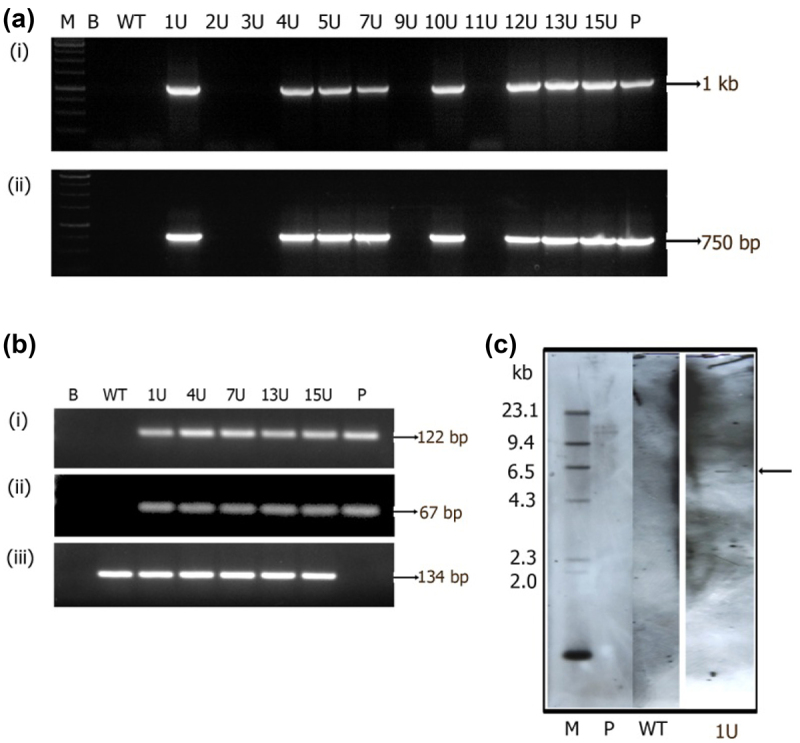
Molecular analysis of primary transformants harboring pCAMBIA 2301 *GUS::nptII*. (a) PCR analysis for the amplification of (i) *GUS* gene (1000 bp) and (ii) *nptII* (750 bp) fragments. Lane M- 1 kb marker, Lane B- water blank, Lane WT- wild-type DNA, Lanes 1 U-12 U- are primary transformants, Lane P- plasmid (25 ng). (b) sqRT-PCR amplified products on 1.5% w/v agarose gel of (i) 122 bp *GUS*, (ii) 67bp *nptII* and (iii) 134 bp *MiACT1*. Lane B- water blank, Lane WT- wild type, Lanes 1 U, 4 U, 7 U, 13 U, 15 U- transgenic plants, Lane P- binary vector. (c) Genomic Southern analysis of transgenic plants probed with DIG-labeled 750 bp *nptII* gene fragment, Lane L- Lambda *Hind*III DNA digest, Lane WT- wild type, Lane 1 U- transgenic Mango, Lane P- linearized plasmid of pCAMBIA2301 (10 pg).

Out of 12 plants tested for *GUS* and *nptII* genes, 8 plants showed PCR amplification of 1 kb *GUS* gene and 750 bp *nptII* gene fragments indicating the presence of transgenes in the primary transformants (Fig. 6a i-ii). Five *GUS* positive PCR plants were further tested for transgene accumulation that have shown amplification of 122 bp (*GUS*) and 67 bp (*nptII*) fragments verifying the presence of transcripts in transgenic plants and their absence in wild-type plants (Fig. 6b i-ii). Amplification of *MiACT11* internal gene fragment in both transgenic and wild-type plants by sqRT-PCR further authenticated the results (Fig. 6b iii). Furthermore, transgene integration by Southern blotting identified single copy integration of the transgene in one transgenic plant (1 U) and the hybridization signal was absent in the wild-type plant DNA precisely confirming the transgenic nature of the plant (Fig. 6c).

Several researchers have tried to develop transformation protocols for mango using different methodologies.^9–12,46,59,60^ However, in all the previous studies they were unable to unequivocally demonstrate transformability, more so the transgene integration by genomic Southern analysis. This study demonstrated gene introgression in mango genome using shoot apical meristem-targeted *in planta* transformation and the associated molecular analyses. Tree crops have always been found difficult to improve by conventional breeding tools, biotechnology complement the conventional breeding and amends the mango improvement programs^48^. Transgenic technology holds several promises and can open ways in tackling a multitude of problems in mango

## 4. Conclusion

This study demonstrates the amenability of mango to apical meristem targeted *in planta* transformation protocol in a genotype independent manner. This report is the first successful demonstration of transgenic mango development using *in planta* transformation, which can assure it to be a significant contribution toward advancement in the area of mango biotechnology.
